# One health approach to tackle brucellosis: a systematic review

**DOI:** 10.1186/s41182-020-00272-1

**Published:** 2020-10-20

**Authors:** Mahboubeh Khaton Ghanbari, Hasan Abolghasem Gorji, Masoud Behzadifar, Nadia Sanee, Nafiul Mehedi, Nicola Luigi Bragazzi

**Affiliations:** 1 Student Research Committee, School of Health Management and Information Sciences, Iran University of Medical Sciences, Tehran, Iran; 2 Zoonoses Control Unit, Center of Diseases Control, Ministry of Health and Medical Education, Tehran, Iran; 3 Health Management and Economics Research Center, Iran University of Medical Sciences, Tehran, Iran; 4 Social Determinants of Health Research Center, Lorestan University of Medical Sciences, Khorramabad, Iran; 5 Department of Social Work, Shahjalal University of Science and Technology, Sylhet, Bangladesh; 6 Department of Health Sciences (DISSAL), Postgraduate School of Public Health, University of Genoa, Genoa, Italy

**Keywords:** One Health, Zoonotic disease, Brucellosis, Emerging and re-emerging infections, Systematic review

## Abstract

**Background:**

Brucellosis is the most significant and common bacterial zoonosis and is recognized as a re-emerging and neglected disease. Tackling zoonosis is very important for the health and the economy. One Health is an approach characterized by the integration of human and animal health, plants, and ecosystems and encourages joining local, national, and global multidisciplinary efforts to achieve optimal levels of health and collaboration among different disciplines to address complex health problems.

**Objectives:**

The present study aimed to review published scientific literature related to the use of the One Health approach to tackle human brucellosis.

**Methods:**

Web of Science (WoS), PubMed, Scopus, The Cochrane Library, and Embase databases were searched from inception until 30 January 2020. The reference lists of all relevant papers were hand-searched. Two authors extracted data from published studies independently. The Joanna Briggs Institute tool was used to assess the quality of studies.

**Results:**

Of 2297 studies, 10 studies were deemed eligible, which were conducted between 2013 and 2019. Studies were performed in Uganda, Malta, Serbia, Greece, Mongolia, Azerbaijan, Israel, India, Ethiopia, and the USA. All studies suggested that brucellosis is still a major public health problem and that the most important aspect of the One Health approach is the interdependence of humans, ecosystems, and animals .Some studies have focused on livestock vaccination as the most effective way to prevent disease, and others have focused on the biology of *Brucella* infection and its transmission patterns. Some studies have pointed to the effectiveness of the One Health approach in all the phases of disease management as well as to its role in reducing health costs.

**Conclusion:**

The success of the approach depends on the willingness of the decision-makers to implement the necessary policies. Due to the heterogeneity of current practices, and organizations involved in One Health approach-based programs, it will be incomplete without proper planning. To better implement the approach, strategies should be appraised and disseminated by experts and relevant stakeholders.

## Background

Zoonoses are transmissible diseases between vertebrate animals and humans. Brucellosis is the most significant and common bacterial zoonosis and is recognized as a re-emerging and neglected zoonotic disease [1, 2]. Tackling zoonosis is very important for the health and the economy. This disease disrupts daily activities as well as decreases livestock production [3]. In terms of the impact on poor people, brucellosis is ranked as the highest and tenth in a study of 76 animal diseases and syndromes, respectively [4]. Brucellosis has been prevalent in many parts of the world, and there is a risk of re-emergence also in countries that have developed an effective disease control and even eradicated the infection [5, 6].

The incidence rate in endemic countries is 10%, and the death rate is low. However, the World Health Organization (WHO) estimates that a quarter of cases are unreported, with only half of a million cases per year being registered as brucellosis. The number of unreported cases with unspecified clinical symptoms is ten times higher. Thus, it is one of the most significant public health concerns [1, 3, 7]. Brucellosis can affect all age and sex groups, and its control in humans depends on limiting the infection in animals through vaccination and care programs [1, 8, 9]. “One Health” is an approach based on the integration of human and animal health, plants, and ecosystems and encourages joint local, national, and global multidisciplinary efforts to achieve optimal levels of health and collaboration between different disciplines to address complex health problems [10–13]. It is crucial to provide new ways and tools to research and execute effective services to support the formulation of norms, regulations, and policies for the benefit of humanity, animals, and the environment for the present and future of generations. As such, it is necessary to understand how to predict, diagnose, prevent, and control infections by strengthening the links among the various health-related domains and by reducing overlaps among the sectors. This can increase the efficiency and cost-effectiveness of health policies and plays a significant role in achieving the Sustainable Development Goals (SDGs), improving equity in the world [12, 14–19]. To use the One Health approach to tackle brucellosis, accurate identification of the sources of infection and development of targeted control strategies in animals are of particular importance. In disease management, there is evidence that a proper approach and effective interventions can result in reduction of brucellosis cases [10, 20]. Capacity building for brucellosis surveillance, management, and treatment program in developing countries face many challenges, and, because of the complex nature of its control, international standards and policies can provide a common framework for planning in the field [15, 21–23]. The purpose of this study is to evaluate the One Health approach to combat brucellosis in different countries, identify gaps in current practices, and provide recommendations.

## Methods

This study was conducted according to the “Preferred Reporting Items for Systematic Reviews and Meta-Analyses” (PRISMA) Guidelines [24] (Appendix 1). The study protocol has been registered within the international registry “Open Science Framework” (OSF; registration code 10.17605/OSF.IO/D4GKQ).

### Search strategy

Web of Science (WoS), PubMed, Scopus, The Cochrane Library, and Embase databases were searched by two authors independently up to 30 January 2020. The reference lists of all relevant papers found electronically were also hand-searched and this enabled us to retrieve further 32 records. The search strategy performed is reported in Appendix 2.

### Study eligibility criteria

#### Inclusion criteria

Inclusion criteria were (i) studies in which the One Health approach is used to investigate programs and policies related to brucellosis, (ii) studies published in a peer-reviewed journal, (iii) studies written in the English language, and (iv) studies not limited to special or exposed populations.

#### Exclusion criteria

Exclusion criteria were (i) studies designed as a letter to editor, editorial, commentary, book chapter, case-reports, or case-series; (ii) studies published in a non-English language; (iii) studies unavailable in full-text; and (iv) studies whose findings were deemed inadequate or insufficient.

### Study selection

Search results were downloaded to EndNote Edition Version 8. After removing duplicate items, two researchers screened the title and abstract of the documents based on the inclusion and exclusion criteria. The two researchers resolved the conflict through negotiations. Otherwise, a third researcher decided whether to include the article in the present study or not.

### Data extraction

The data collection tool was a spreadsheet organized as data extraction form. The research team designed this form that included the major bibliographic characteristics of retained studies as the first author, the publication year, and the place of study. The study design and the main findings related to the study topic were also extracted.

### Study quality

The Joanna Briggs (JB) “Checklist for analytical cross-sectional studies” was used to assess the quality of studies. This checklist was prepared and approved by the JB Institute and is commonly used in systematic review studies. This tool consists of eight questions with 4 possible answers (yes, no, unclear, and not applicable).

## Results

**Fig. 1:**
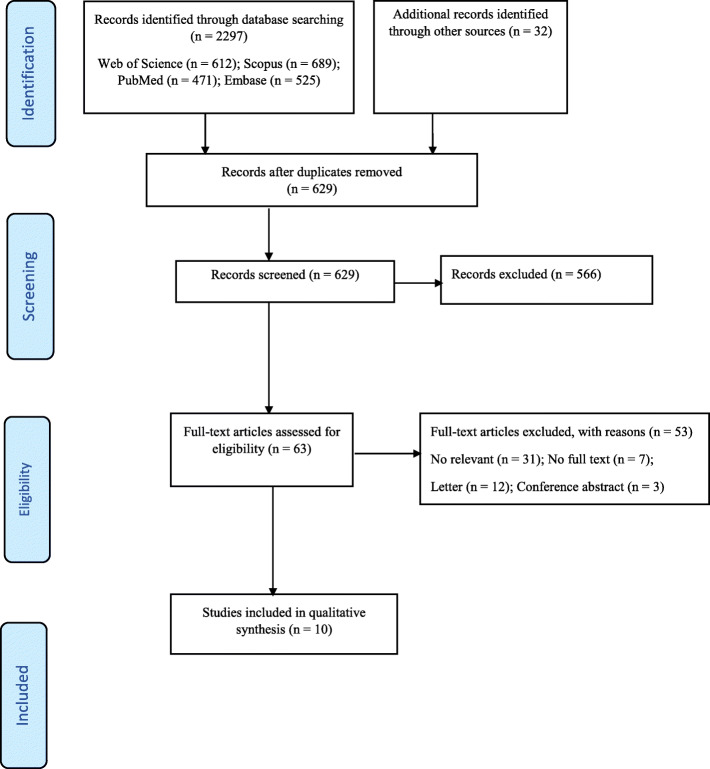
PRISMA flow diagram showing the search, retrieval, and selection of potentially relevant studies

The initial search yielded a pool of 2297 studies. There were 1668 duplicate studies. After removing them, the titles of 629 studies were reviewed; 566 of them were unrelated to the topic and were removed. Abstracts of 63 studies were reviewed, and 53 irrelevant studies were removed. Finally, 10 studies were selected based on inclusion and exclusion criteria. The process of searching and selecting studies is shown in Fig. 1. Table 1 summarizes the characteristics of the 10 studies included in the present study.

**Table 1:** Characteristics of the included studies

First author (reference)	Year of publication	Country	Methods	Main finding
Buttigieg [21]	2018	Malta and Serbia	In this comparative case study, a retrospective comparative study was conducted in Malta and Serbia in 2018 on the Brucellosis Control Program during two time periods: 1995–1997 and 2004–2006. The quantitative assessment of its compliance with the One Health approach was done. It was developed based on the Network for Evaluation of the One Health approach, the framework of which is based on change theory, process evaluation, operational infrastructure, and support and examining the relationship between processes. In the present study, the researchers identified the results of operations and infrastructures of the One Health approach to the control and eradication of brucellosis through a comprehensive evaluation of these aspects.	The results showed that the context and timing are two key factors in determining how, when, and why to use the One Health approach. Therefore, in order to use this approach in potential health crises, one should not seek to fully-fledge it, because each relevant group must be alert and fulfill its key responsibilities in the early stages before interdisciplinary interventions become necessary. Adopting this approach not only in times of crisis but also in the medium and long term, especially in the areas of disease prevention and control, surveillance programs, health promotion, and health education, has also helped save costs and may add value. Therefore, in order to use this approach and evaluate it, economic evaluations should be done so that they can identify the optimal use of resources in these cases and thus justify the necessary budget and political support.
Fouskis [7]	2018	Greece	The present descriptive study was conducted in Greece based on reviewing and updating statistical data of brucellosis over a period of time from 2012 to 2007. In this study, the epidemiological data of human brucellosis were collected not only by determining the incidence disease but also by examining the relationship between human brucellosis and disease in small mammals and estimating its associated risk factors based on the One Health approach. Known risk factors such as direct contact with animals, recent consumption of dairy products, high-risk occupations, and recent travel history, gender, ethnicity, and age group were analyzed. The correlation between vaccination and disease incidence was evaluated	Results of this study showed that brucellosis will remain a significant public health issue and will subsequently affect the Greek agricultural economy, because updated information on brucellosis in Greece revealed seasonal differences and patterns of transmission. There are still brucellosis zones in Greece, so more effective cooperation among the public health departments involved in this issue should be pursued to effectively control brucellosis. There was a statistically significant difference in the incidence of human brucellosis between eradicated and vaccinated zones.
Godfroid [25]	2013	Uganda Mongolia Greece	The present study, a descriptive review, covered topics such as brucellosis control and eradication program, brucellosis serology, mass vaccination against animal brucellosis for human health, various nomadic populations, and brucellosis in animal species and at the animal-human interface.	The researchers concluded that the approach used in brucellosis and other zoonotic diseases should be able to encourage people in medicine, veterinary medicine, wildlife, and sociology to gain a full perception and understanding of the disease. It should also encourage people to engage in professional, scientific, and documentary participation in the formation of collective and effective disease control strategies. For this reason, authors proposed principles for implementing this approach, which included identifying *Brucella* species, paying attention to vaccination status, vaccinating animals, and paying attention as well to other species and the source of its transmission to humans. In order to support accurate control measures in the maintenance host, recognizing the biology of *Brucella* infections and its patterns of transmission in wildlife, as well as between livestock and humans, is of particular importance. Before implementing any control and eradication program, it is necessary to identify *Brucella* species that infect animals, and the necessary interventions should be integrated into the One Health Program. Infected and non-infected animals (both) need to be vaccinated at the time of mass vaccination. Calves, lambs, and piglets born to infected animals may be infected, regardless of their vaccination status, even if they appear to be healthy. These animals, even when vaccination is implemented, maintain infection in the animal population. The contribution of non-conventional livestock species (yacks and camels) to human brucellosis should also be investigated. As vaccines interfere with serology, vaccination status should always be considered, especially when studies rely on seroprevalence of disease. The source of human brucellosis cases is mainly related to food (milk and dairy products) or occupation (farmer, butcher, veterinarian, etc.). If human cases are found mainly in certain occupational categories, it indicates that the health measures related to milk and dairy products are effectively done and control should be strengthened in the animal species of the reservoir. If most cases are found in the general population, it indicates that neither health measures nor control measures are being implemented effectively.
Godfroid [10]	2017	Industrialized and low- and middle-income countries (LMICs)	This review study examined the One Health approach for the control of brucellosis in industrialized, low-income, and middle-income countries. The study assessed whether the standard methods and other health interventions were adequate and ethically sound. The results are also showing the knowledge gap about the biology of *Brucella* infections.	The result of this study showed that understanding the biology of *Brucella* infections and its transmission patterns in wildlife and between animals and humans is of particular importance, and even before any animal control or eradication program is implemented, more intervention should be performed. Experimenting with the One Health plan empirically, the most important aspect of the program is to show the interdependence of humans, ecosystems, and animals in terms of disease and health. However, even if one considers animals morally valuable, one should consider them to be less valuable than humans. So it is justifiable to put them in the interests of future generations of humans and animals. Conversely, if one concludes that slaughtering is not morally justified, even if it avoids animal suffering, slaughtering would be unacceptable. The One Health approach potentially constitutes a paradigm shift in our worldview, forcing to rethink the understanding of the ethical status of animals, plants, and ecosystems.
Gemechu [22]	2017	Ethiopia	This review study examined how to control brucellosis through the One Health approach in Ethiopia. Various aspects of brucellosis in humans and animals, including epidemiology and etiology, have been examined to shed light on the transmission and risk factors of the disease.	It was concluded that veterinary, medical, and environmental groups should work together in a four-pronged approach to identify the potential risk factors for the disease and design appropriate countermeasures. Unfortunately, in many underdeveloped and developing countries, this type of cooperation is either absent or very weak. These issues have provided an opportunity for the development of brucellosis, especially in rural areas, and the elimination of this disease is not possible without considering these issues.
Hermesh [19]	2018	Israel	This qualitative study was aimed to design the role of different stakeholders in the fight against brucellosis in the Negev region of Israel. Authors also examined the political and historical aspects of these actors’ understanding of appropriate interventions in disease control. This study conducted twenty in-depth interviews with policy-makers, human, and animal health professionals, local community representatives. Target population interventions and observations, and documentation review were also done as well as stakeholder knowledge (policy-makers, stakeholders, and livestock owners) also gathered. Understanding of appropriate interventions to control brucellosis was also assessed during interviews. Their perceptions of brucellosis, its nature, causes, job or livelihood status, and cooperation with various institutions to tackle the disease were discussed. Participants were observed in decision-making aspects, such as the Israeli parliament, joint ministry meetings, and meetings hosted by the Israeli Veterinary Service. Media and policy-making documents were collected using relevant information from the Google search engine and the websites of the Israeli Ministry of Health. Also, the benefits of developing the One Health approach for ethical cohesion and its social and political aspects in the control of brucellosis were investigated.	The results showed that incorporating historical, political, and biological considerations of public health into developing the One Health approach provides an opportunity to increase the relevance of this approach and expand its scope as a new scientific paradigm. Because at present, most interventions are based on instrumental efforts to strengthen stakeholder collaboration with specific frontiers in the fields of veterinary, medical, and agricultural sciences, such an approach would require addressing the health discourse and practice of structural inequalities. It was also stated that, although the One Health approach, as an international movement and as a research method, wishes to cross the boundaries between disciplines, nevertheless, due to the over-emphasis on physicians and veterinarians, the capacity of the care program is also known as “reductionist manner.”
Kracalik [9]	2014	Azerbaijan	In a cross-sectional descriptive study conducted in Azerbaijan, authors assessed the annual epidemiological and spatial incidence of human brucellosis using animal care and control program data during the years from 2002 to 2009 from a single health perspective.	The findings showed that the occurrence of human brucellosis has a pattern of re-emergence in Southeast Azerbaijan. It seems the disease was emerging from 1983 to 2009, when a total of 11,233 cases of human brucellosis were reported. Until the mid-1990s, human brucellosis showed a pattern of re-emergence with an average increase of 25% annually. The findings also strengthened the role of animal vaccination in controlling brucellosis and concluded that the One Health approach is needed to address the changing pattern of brucellosis in the Republic of Azerbaijan and elsewhere in the former Soviet Union.
Kaneene [20]	2018	Uganda	This cross-sectional descriptive study was conducted to investigate outbreaks of zoonotic diseases such as brucellosis. The research team consisted of two units (Public Health and Animal Health) that participated in the project implementation. The benefits of using the One Health approach in outbreaks and brucellosis in the human and livestock population in Uganda were recorded and compared with the results of the two units which were working separately.	The main results of the present study included the preparation of a protocol for collecting laboratory samples, the method of transporting them and conducting experiments in the laboratory, the development of training programs for the investigations and research in the field of zoonotic diseases. There was also a program for farmers on how the disease transmits between humans and animals (using the One Health approach) and results and communication were shared between representatives of the Ministry of Health and the Ministry of Agriculture, Animal Industry, Fisheries, and Wildlife. One of the main results of the research was to reduce tensions with the agricultural sector. Therefore, using the One Health approach in research and studies on outbreaks of zoonotic diseases such as brucellosis has several advantages and is much less expensive than conducting two separate studies (one by the Public Health team and the other by the Animal Health Departments).
Lindahl [26]	2019	India	The study, published in India in 2019, describes the results of a joint workshop to determine the One Health approach priorities in the control of brucellosis. The workshop, organized by the International Institute for Livestock Research, is attended by government experts, national research institutes, universities, and invited various international organizations to a 1-day meeting to set out the priorities of a “hygiene strategy” for controlling brucellosis in India. The priority of these strategies includes cooperation (transboundary and cross-sectoral) gathering more epidemiological evidence in humans, cattle, and small ruminants (neglected in past research), economic impact studies (including the cost-effectiveness of control programs). These include vaccination livestock (including national facilities for vaccines for cows), managing infected animals (prohibiting the slaughter of cattle), laboratory capacity and detection (quality and speed of performance), raising awareness (farmers, healthcare workers), and making the general public aware of the dangers of brucellosis and zoonosis in general.	The results of the workshop showed that although India faces many challenges in the control of brucellosis, the success of this initiative depends on cooperation between institutions, neighboring countries, and international institutions. The results of the workshop provide suggestions for joint strategies for the promotion of brucellosis control with a multi-pronged One Health approach that coordinates their performance in both veterinary and medical fields.
Plum b[23]	2013	USA	This study review, conducted in the USA, examined the challenges and opportunities for the One Health approach. In the present study, seven key factors were considered in the One Health approach, which included factors such as medicine, politics, ecology, science, socioeconomics, education, and management.	The study showed that challenges and opportunities must be identified in the management of brucellosis, which is fundamentally multivariate, multifaceted, and integrated. Therefore, it is essential that a brucellosis training curriculum in the form of the One Health approach for the veterinary, public health, and wildlife and environmental protection professions will provide a common framework for interactive training among statesmen and administrators. To prioritize and demonstrate the economic benefits of major investments in brucellosis research, diagnosis, surveillance, and management in human and animal health sectors, developing and analyzing its effects are important. Adaptive risk management (AMR) could provide a framework for supporting stakeholders to address complexities and uncertainties and to learn management practices. The integration of the global One Health approach must be implemented to overcome the under-reporting and underestimation of disease.

Studies were conducted in Uganda, Malta, Serbia, Greece, Mongolia, Azerbaijan, Israel, India, Ethiopia, and the USA. All studies have suggested that brucellosis will continue to be a major public health problem; that the most significant aspect of the One Health approach is to show the interdependence of humans, ecosystems, and animals in terms of disease and health; and that multidisciplinary investigations should be recommended. Various veterinarians, physicians, specialists, and environmentalists should work together within the One Health framework to identify potential risk factors for the disease and to design appropriate countermeasures. Thus, this program will create a common approach for interactive training among government officials, managers, doctors, technicians, and the general public. Some studies have focused on the vaccination of livestock as the most effective way to prevent disease in humans [7, 9, 22, 25, 26], while some studies have focused on the biology of *Brucella* infection and its patterns of transmission in wildlife and between livestock and humans. Even before implementing any animal control or eradication program, more interventions with the One Health program should be performed [9, 10, 25]. Some studies have pointed to this approach in all periods of disease management (crisis, outbreak, medium- and long-term) [9, 20, 21], emphasizing the role of the One Health approach in reducing health costs and even generating cost savings [20–23]. Even though the incidence of the disease in the Third World and developing countries is much higher than in developed ones, not many studies have been conducted in underdeveloped countries, due to lack of financial support for the One Health program and the dearth of disease surveillance programs in some of these countries. As such, the One Health approach should be implemented especially in these settings.

Based on the JBI checklist, the quality of the selected studies and the results of the survey are presented in Table 2.

**Table 2:** Methodological assessment of the quality of selected studies

First author (reference)	Q1	Q2	Q3	Q4	Q5	Q6	Q7	Q8
Buttigieg [20]	Yes	Yes	Yes	Yes	Yes	Yes	Yes	Not applicable
Fouskis [7]	Yes	Yes	Yes	Yes	Yes	Yes	Yes	Yes
Godfroid [25]	Yes	Yes	Yes	Yes	Yes	Yes	Yes	Not applicable
Godfroid [10]	Yes	Yes	Yes	Yes	Yes	Yes	Yes	Not applicable
Gemechu [22]	Yes	No	No	No	No	No	No	Not applicable
Hermesh [19]	Yes	Yes	Yes	Yes	Yes	Yes	Yes	Yes
Kracalik [9]	Yes	No	No	No	No	No	No	Not applicable
Kaneene [20]	Yes	Yes	Yes	Yes	Yes	Yes	Yes	Not applicable
Lindahl [26]	Yes	Yes	Yes	Yes	Yes	Yes	Yes	Not applicable
Plumb [23]	Yes	No	No	No	No	No	No	Not applicable

## Discussion

The present study provides published evidence on the use of the One Health approach to combat brucellosis. Although One Health has been defined since 2000 as a multidisciplinary and international collaborative approach aimed at optimizing the health at the animal-human ecosystem interface, it has been formally adopted only since 2007. The term “One Health” emerged from the joint efforts of the American Veterinary Medical Association (AVMA) and the American Medical Association (AMA) [27, 28]. The findings of this study, which were extracted from ten selected articles, showed that the role of livestock vaccination in the prevention and control of brucellosis is very important and it is almost impossible to control and eradicate the disease without it [7, 9, 26, 28]. There was a statistically significant difference between vaccination and brucellosis incidence in eradicated zones [7, 9]. Moreover, in order to support accurate control measures in the maintenance host, recognizing the biology of *Brucella* infections and its species and patterns of transmission in wildlife, as well as between livestock and humans, is of particular importance.

Before implementing any control and eradication program, it is necessary to identify *Brucella* species that infect animals, and the necessary interventions should be integrated into the One Health program [10, 25]. Both infected and non-infected animals need to be vaccinated at the time of mass vaccination. Calves, lambs, kids, and piglets born from infected animals may be infected too, regardless of their vaccination status, even if they appear to be healthy. These animals, even when vaccination is implemented, maintain infection in the animal population. The contribution of non-conventional livestock species (such as yaks and camels) to human brucellosis should also be investigated. As vaccines interfere with serology, vaccination status should always be considered, especially when studies rely on seroprevalence of disease. The role of preventive vaccination in reducing the abortion rate and *Brucella* excretion in breast milk is well documented.

However, the therapeutic value of animal vaccination (i.e., vaccination of infected animals), in particular, its ability to reduce the number and duration of excretion of *Brucella* spp. in milk, remains debated and further investigations are needed [25]. It should be noted that vaccination alone is not sufficient for success in the prevention and control of brucellosis. Governments should be involved in this issue and should raise the awareness of people of their countries about the risk of the disease. These interventions will be profitable and cost-effective for the agricultural and health sectors if vaccination costs against brucellosis are allocated to all involved sectors in proportion to the benefits [25, 29]. If human cases are found mainly in certain occupational categories, this indicates that the public health control measures related to milk and dairy products have been effectively implemented. Control programs should be strengthened in particular in the animal species of the reservoir. If most cases are found in the general population, this indicates that neither health measures nor control measures have been effectively implemented [25].

The studies retained in the present systematic review provided recommendations for the optimal use and application of the One Health approach. In many underdeveloped and developing countries with high incidence and burden of disease, these substrates are virtually nonexistent or very weak [22, 25]. Therefore, in some countries, such as Ethiopia, which does not have a coherent surveillance program for this disease, this approach should be implemented [22] and governments should provide the conditions for moving toward the One Health approach [7, 9, 25]. This framework is not only suitable for long-term surveillance and control programs, but also useful for the mid-term management for crises and outbreaks, and even in periods when the disease has not emerged yet [20, 21] with various benefits, including cost savings, especially in the areas of disease prevention and control, surveillance programs, health promotion, and health education [20, 21, 23].

In order to implement this approach, the integration of all groups and organizations involved in disease surveillance, including veterinary, medical, and environmental specialists, as well as scholars working in other disciplines such as sociology, is essential. Groups should work together to fully understand the determinants of infectious diseases with the aim of identifying possible risk factors and designing appropriate ways to deal with them, leading to the development and implementation of collective and effective disease control strategies [7, 21–23, 25].

To face potential health crises, all relevant groups should always be vigilant and responsive since the early stages before interdisciplinary interventions are required [21]. Understanding the historical, political, and biological implications of public health within the One Health approach provides an opportunity to increase the relevance of this approach and expand its scope as a new scientific model [19].

At present, most of the public health interventions implemented by governments are based on instrumental efforts to strengthen cooperation between stakeholders with clear boundaries among the fields of veterinary, medical, and agricultural sciences [19, 22, 25]. The approach based on the concept of One Health eliminates, instead, structural inequalities, and “reductionist manner” programs relying on the over-emphasis on physicians and veterinarians [19]. Adaptive risk management (ARM) can provide a proper theoretical framework for supporting stakeholders in addressing the complexities as well as in shifting toward the implementation of effective management practices. This type of acknowledgement-based management deals with uncertainties, provides a dynamic framework for coping with the components of a complex brucellosis control system, and learns from system feedbacks [23]. ARM is a set of possible options that should be dynamically monitored to obtain sufficient information and knowledge about the impact of different performance methods [30]. Economic assessments are also essential for the use, evaluation, and development of this approach.

It is important to prioritize and demonstrate the economic benefits of significant investments in brucellosis research, diagnosis, surveillance, management, and animal health sectors [21]. Being able to identify the optimal use of resources, justifying the necessary budget and having political support is essential. One Health-based programs should be prioritized as dynamic and sustainable rather than conventional ones [21, 23].

The role of education in disease control, by raising awareness of One Health approach among policy-makers, stakeholders, farmers, health care workers, and the general public, is critical to the tackle brucellosis and other zoonoses. Most of the rural population in Asia and Africa have a low level of awareness of brucellosis: understanding the risk of this disease can affect the development and implementation of appropriate disease control strategies as well as the adoption of the best practices [23, 26, 31].

In many countries, the health care program is poorly organized and formal data (obtained passively) underestimates the real burden of the disease. Although it imposes a major burden in the underdeveloped and developing countries, a global approach relying on the concept of One Health should be implemented to curb such a burden [23, 26, 32]. Success in this program depends on cooperation between institutions and agencies within countries and collaboration with neighboring countries [26].

Although all studies have suggested this approach, our results indicate that countries have not yet integrated policies to implement it. The limitations of the present study include the heterogeneity and the different methodologies adopted by the included articles, which hindered a formal quantitative analysis.

## Conclusion

This review presents an up-to-date evidence base for controlling brucellosis within the One Health approach. The success of One Health programs depends on the willingness of statesmen and policy-makers. Due to the fragmented nature of the organizations and stakeholders involved in the issue of brucellosis control, integration among the organizations is required, and programs based on the One Health approach should be prioritized, planned, and implemented.

Because the disease is chronic and has a low mortality rate, usually little attention is paid to control the disease. However, due to the re-emergence of the disease, it also threatens developed countries. In order to better implement the approach, all resources should be mobilized, and all strategies, challenges, and opportunities should be appraised by involving experts and relevant stakeholders. Further research is needed to shed light on the barriers that hinder the adoption of such an approach to prevent and control brucellosis.

## Supplementary information

**Additional file 1.** PRISMA 2009 Checklist

**Additional file 2.** A search strategy in databases

## Data Availability

Not applicable.

## Abbreviations

WHO: World Health Organization
SDGs: Sustainable Development Goals
PRISMA: Preferred Reporting Items for Systematic Reviews and Meta-Analyses
AMA: American Medical Association
AVMA: American Veterinary Medical Association
ARM: Adaptive risk management
JB: Joanna Briggs
