# Supplementary material for: One health approach to tackle brucellosis: a systematic review
Source: Trop Med Health. 2020 Oct 20;48:86. doi: 10.1186/s41182-020-00272-1 (PMC7574566; doi:10.1186/s41182-020-00272-1)
Supplement: Supplementary file 2 — Additional file 2. A search strategy in databases [file 41182_2020_272_MOESM2_ESM.docx]

**Appendix 2: A search strategy in databases**

**Pubmed =147**

- “One health”[tiab] AND (Brucelloses[tiab] OR “malta fever”[tiab] OR (Fever[tiab] AND Malta[tiab]) OR “Gibraltar Fever”[tiab] OR (Fever[tiab] AND Gibraltar[tiab]) OR “Rock Fever”[tiab] OR (Fever[tiab] AND Rock[tiab] OR “Cyprus Fever”[tiab] OR (Fever[tiab] AND Cyprus[tiab]) OR “Brucella Infection”[tiab] OR “Brucella Infections”[tiab] OR (Infection[tiab] AND Brucella[tiab]) OR “Undulant Fever”[tiab] OR (Fever[tiab] AND Undulant[tiab]) OR (Brucellosis[tiab] AND Pulmonary[tiab]) OR (Brucelloses[tiab] AND Pulmonary[tiab]) OR “Pulmonary Brucelloses”[tiab] OR “Pulmonary Brucellosis”[tiab])=4000
- "One health”[tiab] AND (Brucelloses[tiab] OR “malta fever”[tiab] OR (Fever[tiab] AND Malta[tiab]) OR “Gibraltar Fever”[tiab] OR (Fever[tiab] AND Gibraltar[tiab]) OR “Rock Fever”[tiab] OR (Fever[tiab] AND Rock[tiab]) OR “Cyprus Fever”[tiab] OR (Fever[tiab] AND Cyprus[tiab]) OR “Brucella Infection”[tiab] OR “Brucella Infections”[tiab] OR (Infection[tiab] AND Brucella[tiab]) OR “Undulant Fever”[tiab] OR (Fever[tiab] AND Undulant[tiab]))=13
- "One health”[tiab] AND (Brucelloses[tiab] OR “malta fever”[tiab] OR (Fever[tiab] AND Malta[tiab]) OR “Gibraltar Fever”[tiab] OR (Fever[tiab] AND Gibraltar[tiab]) OR “Rock Fever”[tiab] OR (Fever[tiab] AND Rock[tiab]) OR “Cyprus Fever”[tiab] OR (Fever[tiab] AND Cyprus[tiab]) OR “Brucella Infection”[tiab] OR “Brucella Infections”[tiab] OR (Infection[tiab] AND Brucella[tiab]) OR “Undulant Fever”[tiab] OR (Fever[tiab] AND Undulant[tiab]) OR “zoonotic disease”[tiab])=147
- "One health”[tiab] AND (Brucelloses[tiab] OR “malta fever”[tiab] OR (Fever[tiab] AND Malta[tiab]) OR “Gibraltar Fever”[tiab] OR (Fever[tiab] AND Gibraltar[tiab]) OR “Rock Fever”[tiab] OR (Fever[tiab] AND Rock[tiab]) OR “Cyprus Fever”[tiab] OR (Fever[tiab] AND Cyprus[tiab]) OR “Brucella Infection”[tiab] OR “Brucella Infections”[tiab] OR (Infection[tiab] AND Brucella[tiab]) OR “Undulant Fever”[tiab] OR (Fever[tiab] AND Undulant[tiab]) OR “zoonotic disease”[mh])=13
- "One health”[tiab] AND (Brucelloses[tiab] OR “malta fever”[tiab] OR (Fever[tiab] AND Malta[tiab]) OR “Gibraltar Fever”[tiab] OR (Fever[tiab] AND Gibraltar[tiab]) OR “Rock Fever”[tiab] OR (Fever[tiab] AND Rock[tiab]) OR “Cyprus Fever”[tiab] OR (Fever[tiab] AND Cyprus[tiab]) OR “Brucella Infection”[tiab] OR “Brucella Infections”[tiab] OR (Infection[tiab] AND Brucella[tiab]) OR “Undulant Fever”[tiab] OR (Fever[tiab] AND Undulant[tiab]) OR “zoonotic disease”[tiab] OR “emerging and reemerging infectious”[tiab]).
- "One health”[tiab] AND (Brucelloses[tiab] OR “malta fever”[tiab] OR (Fever[tiab] AND Malta[tiab]) OR “Gibraltar Fever”[tiab] OR (Fever[tiab] AND Gibraltar[tiab]) OR “Rock Fever”[tiab] OR (Fever[tiab] AND Rock[tiab]) OR “Cyprus Fever”[tiab] OR (Fever[tiab] AND Cyprus[tiab]) OR “Brucella Infection”[tiab] OR “Brucella Infections”[tiab] OR (Infection[tiab] AND Brucella[tiab]) OR “Undulant Fever”[tiab] OR (Fever[tiab] AND Undulant[tiab]) OR “Zoonotic Infections”[tiab] OR (Infection[tiab] AND Zoonotic[tiab]) OR (Infections[tiab] AND Zoonotic[tiab]) OR “Zoonotic Infection”[tiab] OR “Zoonotic Infectious Diseases”[tiab] OR (Disease[tiab] AND “Zoonotic Infectious”[tiab]) OR (Diseases[tiab] AND “Zoonotic Infectious”[tiab]) OR (“Infectious Disease”[tiab] AND Zoonotic[tiab]) OR (“Infectious Diseases”[tiab] AND Zoonotic[tiab]) OR “Zoonotic Infectious Disease”[tiab] OR “Zoonotic Diseases”[tiab] OR (Disease[tiab] AND Zoonotic[tiab]) OR (Diseases[tiab] AND Zoonotic[tiab]) OR “Zoonotic Disease”[tiab] OR “emerging infectious”[tiab] OR “reemerging infectious”[tiab])=461

**Embase=525**

- "One health”:ti,ab AND (Brucelloses:ti,ab OR “malta fever”:ti,ab OR (Fever:ti,ab AND Malta:ti,ab) OR “Gibraltar Fever”:ti,ab OR (Fever:ti,ab AND Gibraltar:ti,ab) OR “Rock Fever”:ti,ab OR (Fever:ti,ab AND Rock:ti,ab) OR “Cyprus Fever”:ti,ab OR (Fever:ti,ab AND Cyprus:ti,ab) OR “Brucella Infection”:ti,ab OR “Brucella Infections”:ti,ab OR (Infection:ti,ab AND Brucella:ti,ab) OR “Undulant Fever”:ti,ab OR (Fever:ti,ab AND Undulant:ti,ab) OR “Zoonotic Infections”:ti,ab OR (Infection:ti,ab AND Zoonotic:ti,ab) OR (Infections:ti,ab AND Zoonotic:ti,ab) OR “Zoonotic Infection”:ti,ab OR “Zoonotic Infectious Diseases”:ti,ab OR (Disease:ti,ab AND “Zoonotic Infectious”:ti,ab) OR (Diseases:ti,ab AND “Zoonotic Infectious”:ti,ab) OR (“Infectious Disease”:ti,ab AND Zoonotic:ti,ab) OR (“Infectious Diseases”:ti,ab AND Zoonotic:ti,ab) OR “Zoonotic Infectious Disease”:ti,ab OR “Zoonotic Diseases”:ti,ab OR (Disease:ti,ab AND Zoonotic:ti,ab) OR (Diseases:ti,ab AND Zoonotic:ti,ab) OR “Zoonotic Disease”:ti,ab OR “emerging infectious”:ti,ab OR “reemerging infectious”:ti,ab)

**Scopus=689**

TITLE-ABS-KEY("One health”) AND (TITLE-ABS-KEY(Brucelloses) OR TITLE-ABS-KEY(“malta fever”) OR (TITLE-ABS-KEY(Fever) AND TITLE-ABS-KEY(Malta)) OR TITLE-ABS-KEY(“Gibraltar Fever”) OR (TITLE-ABS-KEY(Fever) AND TITLE-ABS-KEY(Gibraltar)) OR TITLE-ABS-KEY(“Rock Fever”)OR (TITLE-ABS-KEY(Fever) AND TITLE-ABS-KEY(Rock)) OR TITLE-ABS-KEY(“Cyprus Fever”) OR (TITLE-ABS-KEY(Fever) AND TITLE-ABS-KEY(Cyprus)) OR TITLE-ABS-KEY(“Brucella Infection”) OR TITLE-ABS-KEY(“Brucella Infections”) OR (TITLE-ABS-KEY(Infection) AND TITLE-ABS-KEY(Brucella)) OR TITLE-ABS-KEY(“Undulant Fever”) OR (TITLE-ABS-KEY(Fever) AND TITLE-ABS-KEY(Undulant)) OR TITLE-ABS-KEY(“Zoonotic Infections”) OR (TITLE-ABS-KEY(Infection) AND TITLE-ABS-KEY(Zoonotic)) OR (TITLE-ABS-KEY(Infections) AND TITLE-ABS-KEY(Zoonotic)) OR TITLE-ABS-KEY(“Zoonotic Infection”) OR TITLE-ABS-KEY(“Zoonotic Infectious Diseases”) OR (TITLE-ABS-KEY(Disease) AND TITLE-ABS-KEY(“Zoonotic Infectious”)) OR (TITLE-ABS-KEY(Diseases) AND TITLE-ABS-KEY(“Zoonotic Infectious”)) OR (TITLE-ABS-KEY(“Infectious Disease”) AND TITLE-ABS-KEY(Zoonotic)) OR (TITLE-ABS-KEY(“Infectious Diseases”) AND TITLE-ABS-KEY(Zoonotic)) OR TITLE-ABS-KEY(“Zoonotic Infectious Disease”) OR TITLE-ABS-KEY(“Zoonotic Diseases”) OR (TITLE-ABS-KEY(Disease) AND TITLE-ABS-KEY(Zoonotic)) OR (TITLE-ABS-KEY(Diseases) AND TITLE-ABS-KEY(Zoonotic)) OR TITLE-ABS-KEY(“Zoonotic Disease”) OR TITLE-ABS-KEY(“emerging infectious”) OR TITLE-ABS-KEY(“reemerging infectious”))

**Web of science=612**

TS=("One health”) AND (TS=(Brucelloses) OR TS=(“malta fever”) OR (TS=(Fever) AND TS=(Malta)) OR TS=(“Gibraltar Fever”) OR (TS=(Fever) AND TS=(Gibraltar)) OR TS=(“Rock Fever”)OR (TS=(Fever) AND TS=(Rock)) OR TS=(“Cyprus Fever”) OR (TS=(Fever) AND TS=(Cyprus)) OR TS=(“Brucella Infection”) OR TS=(“Brucella Infections”) OR (TS=(Infection) AND TS=(Brucella)) OR TS=(“Undulant Fever”) OR (TS=(Fever) AND TS=(Undulant)) OR TS=(“Zoonotic Infections”) OR (TS=(Infection) AND TS=(Zoonotic)) OR (TS=(Infections) AND TS=(Zoonotic)) OR TS=(“Zoonotic Infection”) OR TS=(“Zoonotic Infectious Diseases”) OR (TS=(Disease) AND TS=(“Zoonotic Infectious”)) OR (TS=(Diseases) AND TS=(“Zoonotic Infectious”)) OR (TS=(“Infectious Disease”) AND TS=(Zoonotic)) OR (TS=(“Infectious Diseases”) AND TS=(Zoonotic)) OR TS=(“Zoonotic Infectious Disease”) OR TS=(“Zoonotic Diseases”) OR (TS=(Disease) AND TS=(Zoonotic)) OR (TS=(Diseases) AND TS=(Zoonotic)) OR TS=(“Zoonotic Disease”) OR TS=(“emerging infectious”) OR TS=(“reemerging infectious”))

**Cochrane=0**

"One health” AND (Brucelloses OR “malta fever” OR (Fever AND Malta) OR “Gibraltar Fever” OR (Fever AND Gibraltar) OR “Rock Fever” OR (Fever AND Rock) OR “Cyprus Fever” OR (Fever AND Cyprus) OR “Brucella Infection” OR “Brucella Infections” OR (Infection AND Brucella) OR “Undulant Fever” OR (Fever AND Undulant) OR “Zoonotic Infections” OR (Infection AND Zoonotic) OR (Infections AND Zoonotic) OR “Zoonotic Infection” OR “Zoonotic Infectious Diseases” OR (Disease AND “Zoonotic Infectious”) OR (Diseases AND “Zoonotic Infectious”) OR (“Infectious Disease” AND Zoonotic) OR (“Infectious Diseases” AND Zoonotic) OR “Zoonotic Infectious Disease” OR “Zoonotic Diseases” OR (Disease AND Zoonotic) OR (Diseases AND Zoonotic) OR “Zoonotic Disease” OR “emerging infectious” OR “reemerging infectious”)
